# Central European Group on Genetics of Movement Disorders

**DOI:** 10.1111/ene.16165

**Published:** 2023-12-07

**Authors:** Miriam Ostrozovicova, Petr Dusek, Milan Grofik, Vladimir Han, Petr Holly, Robert Jech, Peter Klivenyi, Norbert Kovacs, Kristina Kulcsarova, Egon Kurca, Alexandra Lackova, Veronika Magocova, Jan Necpal, David Pinter, Evzen Ruzicka, Tereza Serranova, Katarzyna Smilowska, Igor Straka, Tatiana Svorenova, Gertrud Tamas, Peter Valkovic, Katerina Zarubova, Henry Houlden, Mie Rizig, Matej Skorvanek

**Affiliations:** ^1^ Department of Neurology P.J. Safarik University Kosice Slovak Republic; ^2^ Department of Neurology University Hospital of L. Pasteur Kosice Slovak Republic; ^3^ Department of Neuromuscular Diseases UCL Queen Square Institute of Neurology London UK; ^4^ Department of Neurology and Centre of Clinical Neuroscience, First Faculty of Medicine Charles University and General University Hospital in Prague Prague Czech Republic; ^5^ Department of Neurology Comenius University and University Hospital Martin Martin Slovak Republic; ^6^ Department of Neurology University of Szeged Szeged Hungary; ^7^ Department of Neurology and HUN‐REN–PTE Clinical Neuroscience MR Research Group University of Pecs, Medical School Pécs Hungary; ^8^ Department of Clinical Neurosciences, Scientific Park MEDIPARK P. J. Safarik University Kosice Slovak Republic; ^9^ Department of Neurology Zvolen Hospital Zvolen Slovak Republic; ^10^ Department of Neurology Silesian Centre of Neurology Katowice Katowice Poland; ^11^ Second Department of Neurology, Comenius University in Bratislava Faculty of Medicine University Hospital Bratislava Bratislava Slovak Republic; ^12^ Department of Neurology Semmelweis University Budapest Hungary; ^13^ Institute of Normal and Pathological Physiology, Centre of Experimental Medicine Slovak Academy of Sciences Bratislava Slovak Republic; ^14^ Department of Neurology, Second Faculty of Medicine Charles University and Motol University Hospital Prague Czech Republic

Over the last two decades, we have witnessed major developments in neurogenetic research, the aetiopathogenesis and possible treatment options. Although substantial progress has been made, the first genome‐wide studies have explained relatively little of the heritability of most complex traits, leading to a theory that a more fine‐scale analysis considering the characteristics of individual populations may hold the key to the missing heritability.

In contrast to the Western part of Europe, where the genetics of movement disorders have been thoroughly studied, genetic reports from patients of Central European ancestry have been scarce as cohorts from this region were rarely included and their genetic background remains unknown. Therefore, the Central European Group on Genetics of Movement Disorders (CEGEMOD) has been established to address this gap, representing a collaboration between nine tertiary movement disorder centres from Slovakia, Czech Republic, Poland and Hungary (Figure 1 and Appendix). The CEGEMOD consortium aims to create a collaborative network that will establish a Central European based registry of movement disorder patients, with focus on Parkinson's disease (PD): atypical parkinsonism such as multiple system atrophy, progressive supranuclear palsy, Lewy body dementia and corticobasal syndrome; prodromal PD (with and without rapid eye movement sleep behaviour disorder) [1] and ataxia. The emphasis is on building a framework for future collaborative studies that will lead to the identification of genetic risk factors and to explore the genotype–phenotype correlations (disease subtypes, age at onset, motor and non‐motor symptoms), leading to the investigation of new, population‐based diagnostic and therapeutic interventions. To achieve this goal, we have established a partnership with the Institute of Neurology, University College London, UK, for their technical support to enable the development of local facilities through education and training.

**FIGURE 1:**
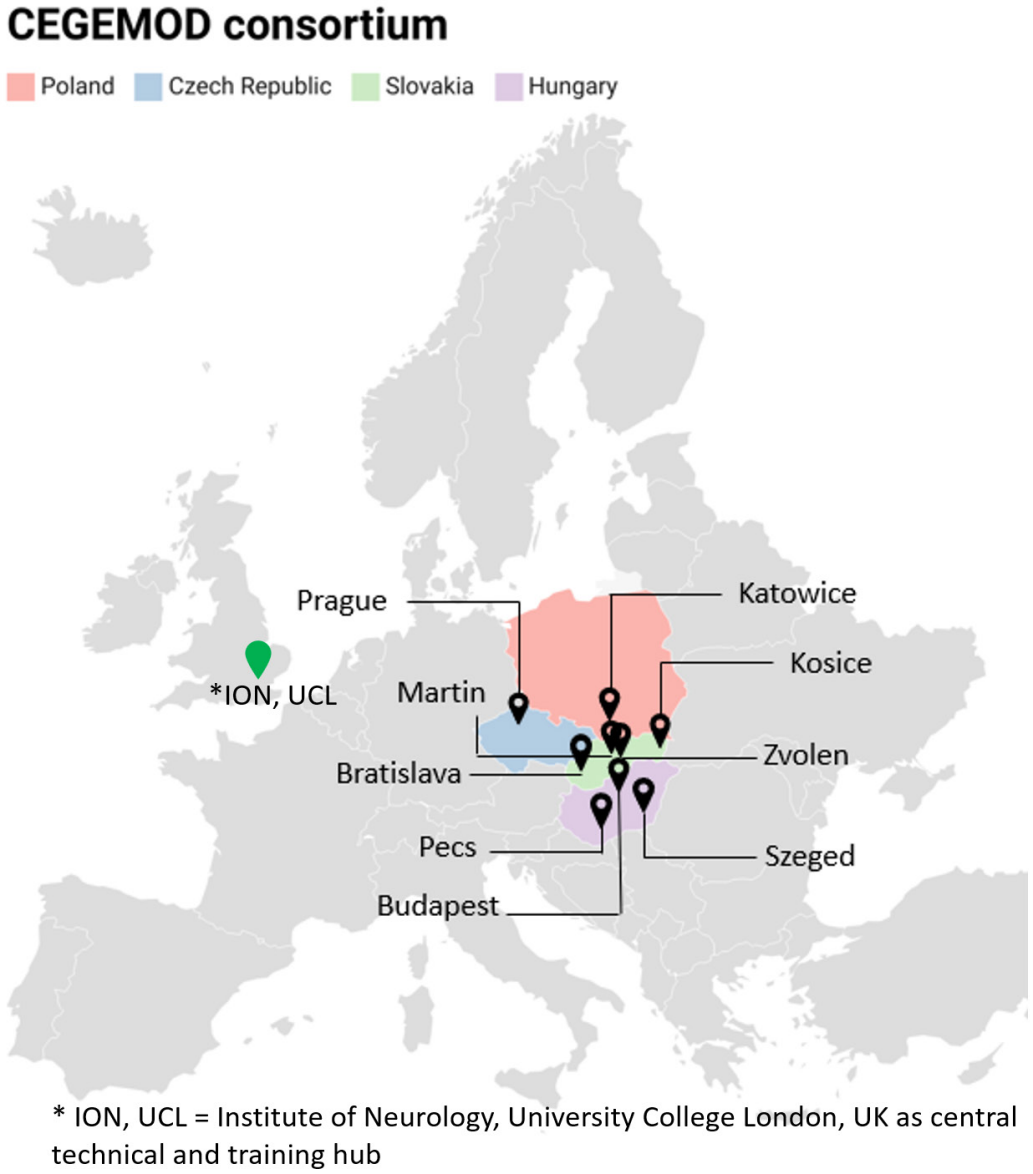
Central European Group on Genetics of Movement Disorders (CEGEMOD). *UCL, ION, Institute of Neurology, University College London, UK, as central technical training hub.

To date, nearly 3500 individuals have been enrolled, making our consortium the largest ethnically matched case–control cohort of Central Europe (Table S1). Each site uses uniform diagnostic criteria and collects a standardized set of clinical and demographic variables.

Our pilot study assessing the contribution of rare *LRRK2* pathogenic variants to PD confirmed that most *LRRK2* variants which are pathogenic in PD in North and West Europe are not a frequent cause of PD in Central Europe. Additionally, we identified a c.1256C>T (p.Ala419Val) *LRRK2* variant that is highly population‐specific and mostly observed in South and East Asians, in two PD patients of Hungarian origin [2]. These findings highlight the potential difference of ethnic background across European countries considering migration patterns, settled ethnic minorities and their genetic influence. The largest ethnic minority of Central Europe, the Roma population, has already been shown to have a common South Asian genetic origin [3] and to harbour several rare disease‐causing variants, although a genetic background of parkinsonism and ataxia specifically has never been studied in this population. Correspondingly, in a related project aimed at the genetics of dystonia, we have already identified two novel founder mutations in this population—the *VPS16* c.559C>T (p.Arg187*) recurrent variant present only in the Roma population [4] and the *WARS2* exon 2 deletion reported only in subjects of Slovak origin so far [5].

## AUTHOR CONTRIBUTIONS

**Miriam Ostrozovicova:** Conceptualization; project administration; writing – original draft; investigation. **Petr Dusek:** Conceptualization; investigation; resources; writing – review and editing. **Milan Grofik:** Conceptualization; investigation; resources; writing – review and editing. **Vladimir Han:** Conceptualization; investigation; writing – review and editing; resources. **Petr Holly:** Conceptualization; investigation; writing – review and editing; resources. **Robert Jech:** Conceptualization; investigation; resources; writing – review and editing. **Peter Klivenyi:** Conceptualization; investigation; resources; writing – review and editing. **Norbert Kovacs:** Conceptualization; investigation; resources; writing – review and editing. **Kristina Kulcsarova:** Conceptualization; investigation; resources; writing – review and editing. **Egon Kurca:** Conceptualization; investigation; resources; writing – review and editing. **Alexandra Lackova:** Conceptualization; investigation; resources; writing – review and editing. **Veronika Magocova:** Conceptualization; investigation; resources; writing – review and editing. **Jan Necpal:** Conceptualization; investigation; writing – review and editing; resources. **David Pinter:** Investigation; conceptualization; writing – review and editing; resources. **Evzen Ruzicka:** Conceptualization; investigation; resources; writing – review and editing. **Tereza Serranova:** Conceptualization; resources; writing – review and editing; investigation. **Katarzyna Smilowska:** Conceptualization; investigation; resources; writing – review and editing. **Igor Straka:** Conceptualization; investigation; resources; writing – review and editing. **Tatiana Svorenova:** Conceptualization; investigation; resources; writing – review and editing. **Gertrud Tamas:** Conceptualization; investigation; resources; writing – review and editing. **Peter Valkovic:** Conceptualization; investigation; resources; writing – review and editing. **Katerina Zarubova:** Conceptualization; investigation; resources; writing – review and editing. **Henry Houlden:** Writing – review and editing; conceptualization; project administration; supervision; resources. **Mie Rizig:** Conceptualization; project administration; supervision; resources; writing – original draft. **Matej Skorvanek:** Conceptualization; writing – original draft; project administration; funding acquisition; investigation; resources.

## FUNDING INFORMATION

This study was funded from the Slovak Grant and Development Agency under contracts APVV‐18‐0547 and APVV‐22‐0279 and by the Slovak Scientific Grant Agency under contract VEGA 1/0712/22; it was also supported by the Operational Programme Integrated Infrastructure, funded by the ERDF (ITMS2014+:313011V455). The Czech Centre was supported by the National Institute for Neurological Research (Programme EXCELES, ID Project No. LX22NPO5107), funded by the European Union, Next Generation EU, and by the Czech Health Research Council grant NU21‐04‐00535. P.K. (Szeged Centre) was supported by TKP‐2021‐EGA‐32.

## CONFLICT OF INTEREST STATEMENT

None of the authors have a conflict of interest to disclose.

## Supporting information


Table S1

## Data Availability

Data sharing not applicable to this article as no datasets were generated or analysed during the current study.

## MEMBERS BY COUNTRY

### Czech Republic

Dusek Petr, Department of Neurology and Centre of Clinical Neuroscience, First Faculty of Medicine, Charles University and General University Hospital in Prague, Prague, Czech Republic. petr.dusek@vfn.cz

Holly Petr, Department of Neurology and Centre of Clinical Neuroscience, First Faculty of Medicine, Charles University and General University Hospital in Prague, Prague, Czech Republic. petr.holly@vfn.cz

Jech Robert, Department of Neurology and Centre of Clinical Neuroscience, First Faculty of Medicine, Charles University and General University Hospital in Prague, Prague, Czech Republic. jech@cesnet.cz

Ruzicka Evzen, Department of Neurology and Centre of Clinical Neuroscience, First Faculty of Medicine, Charles University and General University Hospital in Prague, Prague, Czech Republic. evzen.ruzicka@lf1.cuni.cz

Serranova Tereza, Department of Neurology and Centre of Clinical Neuroscience, First Faculty of Medicine, Charles University and General University Hospital in Prague, Prague, Czech Republic. tereza.serranova@lf1.cuni.cz

Zarubova Katerina, Department of Neurology, Second Faculty of Medicine, Charles University and Motol University Hospital, Prague, Prague, Czech Republic. katzar@centrum.cz

### Hungary

Klivenyi Peter, Department of Neurology, University of Szeged, Szeged, Hungary. klivenyi.peter@med.u-szeged.hu

Kovacs Norbert, University of Pecs, Medical School, Department of Neurology and HUN‐REN–PTE Clinical Neuroscience MR Research Group. kovacsnorbert06@gmail.com

Pinter David, University of Pecs, Medical School, Department of Neurology and HUN‐REN–PTE Clinical Neuroscience MR Research Group. david_pinter@outlook.com

Tamas Gertrud, Department of Neurology, Semmelweis University, Budapest, Hungary.

tamas.gertrud@med.semmelweis-univ.hu

### Poland

Smilowska Katarzyna, Department of Neurology Silesian Centre of Neurology, Katowice, Poland. kasia.smilowska@gmail.com

### Slovak Republic

Grofik Milan, Department of Neurology, Commenius University and University Hospital Martin, Martin, Slovak Republic. milangrofik@gmail.com

Han Vladimir, Department of Neurology, Safarik University and L. Pasteur University Hospital Kosice, Slovak Republic. vladimir.han@gmail.com

Kulcsarova Kristina, Department of Neurology, Safarik University and L. Pasteur University Hospital Kosice, Slovak Republic. kristina.kulcsarova@upjs.sk

Kurca Egon, Department of Neurology, Commenius University and University Hospital Martin, Martin, Slovak Republic. egonkurca@gmail.com

Lackova Alexandra, Department of Neurology, Safarik University and L. Pasteur University Hospital Kosice, Slovak Republic. alexandra.mosejova@gmail.com

Magocova Veronika, Department of Neurology, Safarik University and L. Pasteur University Hospital Kosice, Slovak Republic. veronikamagocova.a@gmail.com

Necpal Jan, Department of Neurology, Zvolen Hospital, Zvolen, Slovak Republic. necpal.neuro@gmail.com

Ostrozovicova Miriam, Department of Neurology, Safarik University and L. Pasteur University Hospital Kosice, Slovak Republic. miriam.ostrozovicova@gmail.com; m.ostrozovicova@ucl.ac.uk

Skorvanek Matej, Department of Neurology, Safarik University and L. Pasteur University Hospital Kosice, Slovak Republic. mskorvanek@gmail.com

Straka Igor, Second Department of Neurology, Comenius University in Bratislava Faculty of Medicine, University Hospital Bratislava, Bratislava, Slovak Republic. straka0105@gmail.com

Svorenova Tatiana, Department of Neurology, Safarik University and L. Pasteur University Hospital Kosice, Slovak Republic. talorinc@gmail.com

Valkovic Peter, Second Department of Neurology, Comenius University in Bratislava Faculty of Medicine, University Hospital Bratislava, Bratislava, Slovak Republic; Institute of Normal and Pathological Physiology, Centre of Experimental Medicine, Slovak Academy of Sciences, Bratislava, Slovak Republic peter.valkovic@gmail.com

### United Kingdom

Houlden Henry, Department of Neuromuscular Diseases, UCL Queen Square Institute of Neurology, London, UK. h.houlden@ucl.ac.uk

Rizig Mie, Department of Neuromuscular Diseases, UCL Queen Square Institute of Neurology, London, UK. m.rizig@ucl.ac.uk
