# Supplementary material for: Central European Group on Genetics of Movement Disorders
Source: Eur J Neurol. 2023 Dec 7;31(4):e16165. doi: 10.1111/ene.16165 (PMC11236003; doi:10.1111/ene.16165)
Supplement: Supplementary file 1 — Table S1 [file ENE-31-e16165-s001.docx]

**Supplementary Table 1** Clinical and demographic characteristics of the cohort (recruited till 08/23).

| **Tertiary Movement Disorder Centre** | **PD** | **Atypical parkinsonism** | **Prodromal PD**  **with RBD / without RBD** | **Ataxia** | **HC** |
| --- | --- | --- | --- | --- | --- |
| **Kosice** | 490 | 64 | 35 / 10 | 44 | 233 |
| **Bratislava** | 178 | 11 | 0 | 0 | 193 |
| **Zvolen** | 176 | 39 | 0 | 7 | 120 |
| **Martin** | 189 | 14 | 0 | 0 | 175 |
| **Prague** | 309 | 15 | 0 | 0 | 277 |
| **Katowice** | 98 | 0 | 0 | 8 | 50 |
| **Budapest** | 165 | 0 | 0 | 0 | 202 |
| **Pecs** | 225 | 14 | 0 | 4 | 12 |
| **Szeged** | 51 | 0 | 0 | 0 | 0 |
| **Total** | **1876** | **157** | **35 / 10** | **63** | **1262** |

PD = Parkinson’s disease; RBD = Rapid eye movement (REM) sleep behaviour disorder; HC = healthy control
